# Treatment of asymptomatic tuberculosis: Protocol for a systematic review of treatment strategies, regimens, and clinical outcomes

**DOI:** 10.1371/journal.pone.0342929

**Published:** 2026-03-02

**Authors:** Yang Li, Ammar Saad, Zhen Feng, Shijia Ge, Lingyun Song, Yanfei Ren, Yan Miao, Ting Li, Tingting Zhao, Wenting Huang, Jiqin Wu, Feng Sun, Xiaolin Wei, Wenhong Zhang

**Affiliations:** 1 Department of Infectious Diseases, Shanghai Key Laboratory of Infectious Diseases and Biosafety Emergency Response, National Medical Center for Infectious Diseases, Huashan Hospital, Shanghai Medical College, Fudan University, Shanghai, China; 2 Shanghai Sci-Tech Inno Center for Infection & Immunity, Shanghai, China; 3 Dalla Lana School of Public Health, University of Toronto, Toronto, Ontario, Canada; 4 National Clinical Research Center for Aging and Medicine, Huashan Hospital, Fudan University, Shanghai, China; 5 Key Laboratory of Medical Molecular Virology (MOE/MOH), Shanghai Medical College, Fudan University, Shanghai, China; Gulu University, UGANDA

## Abstract

**Introduction:**

Asymptomatic tuberculosis (TB) is increasingly recognised through active case finding and prevalence surveys. However, treatment strategies for this patient group remain poorly defined, and there is no consensus on optimal regimens, duration, or outcomes.

**Methods and analysis:**

We will conduct a systematic review of observational and interventional studies reporting anti-TB treatment in patients with asymptomatic TB. Databases including MEDLINE, Embase, Scopus, Web of Science, CINAHL, and the Cochrane Library will be searched without language restrictions. Outcomes of interest include favourable outcome rate, treatment coverage, success rate, relapse, mortality, loss to follow-up, adverse events, and patient-reported outcomes such as pill burden and treatment discontinuation. Study selection, data extraction, and quality assessment will be performed independently by two reviewers, following PRISMA guidelines. Where appropriate, meta-analyses will be conducted using a random-effects model.

**Discussion:**

This review will provide a comprehensive synthesis of current evidence on the treatment of asymptomatic TB. It aims to inform future clinical and public health strategies, including the potential for simplified or shortened treatment regimens tailored to this population. By identifying gaps and inconsistencies in current practice, the findings will support evidence-based decision-making and contribute to global TB control efforts.

## Introduction

Tuberculosis (TB) remains a major global public health threat. In 2023, approximately 10.8 million people fell ill with TB, and 1.25 million deaths were related to TB worldwide, which underscore that TB, has once again become the leading cause of death from a single infectious disease [1].

Recent advances in immunology and microbiology permit new perspectives on the spectrum of TB. Historically, TB has been classified using a binary framework, distinguishing latent TB infection (LTBI) from active TB disease. However, accumulating evidence has suggested that disease progression does not occur as an abrupt transition between these two states. Instead, many individuals experience an intermediate phase in which microbiological or radiological abnormalities are present in the absence of classical TB-related symptoms. This state has been described as asymptomatic or subclinical TB [2,3], and has drawn considerable attention in recent years [3,4]. Reflecting this evolving understanding, the World Health Organization (WHO) has recently reached a consensus that subclinical TB, now referred to as asymptomatic TB, is defined as a person with TB disease who did not report symptoms suggestive of TB during screening, whether or not bacteriologically confirmed [5].

A recent systematic review by Stuck et al. highlights that a large number of active TB cases with less extensive disease may initially present without symptoms, yet still harbour viable *Mycobactrium tuberculosis* and potentially contribute to transmission [6]. This hidden burden presents a major challenge for TB control programmes, as symptom-based screening strategies are unlikely to identify these individuals. From a research perspective, the absence of symptoms complicates the characterization of disease progression and raises important questions regarding the optimal timing and appropriateness of treatment. Improved characterization of this disease state not only deepens our understanding of this previously under-recognized population but also provides critical insights into the natural history of progression from latent infection to symptomatic active disease.

There is an emerging need to distinguish between asymptomatic and symptomatic individuals with TB as their healthcare pathways and management strategies differ substantially in both clinical and public health settings [7]. For individuals with TB who actively seek care due to symptoms, there is broad consensus among patients, physicians, and public health practitioners regarding their classification and treatment. These individuals are universally considered infectious and require the standard anti-TB treatment with regular follow-up. However, perceptions of individuals with asymptomatic TB vary among different stakeholders. Asymptomatic individuals may not perceive themselves as ill due to the absence of symptoms. Physicians, on the other hand, might classify them as having active TB and initiate standard treatment regimens, particularly given that bacteriological confirmation is not always obtained even in symptomatic cases. Additionally, public health practitioners play a role in identifying asymptomatic TB cases through screening and prevalence surveys. However, ensuring successful referral to healthcare facilities for definitive diagnosis remains a challenge, which increase the risk of loss to follow-up [8].

In such a complex context, the current treatment status of asymptomatic TB remains unclear. Previous systematic reviews have provided a detailed description of its definition and epidemiological characteristics [9]. In the available literature, individuals with asymptomatic TB have generally been treated using standard TB treatment regimens. Reported treatment success rates among people with asymptomatic TB appear comparable to those observed in patients with symptomatic TB [9]. However, gaps persist regarding its treatment and prognosis, as existing evidence remains fragmented and has not been systematically synthesized. Therefore, this study aims to analyse treatment regimens, duration, success rates, safety, and long-term prognosis for this population, providing scientific evidence to optimize future treatment strategies.

## Methods

### Registration and results reporting

The study protocol for this systematic review was designed and reported in accordance with the Preferred Reporting Items for Systematic Reviews and Meta-Analysis (PRISMA) extension statement [10,11]. The protocol was prospectively registered in the International Prospective Register of Systematic Reviews (PROSPERO; Registration No. CRD420251005791). The review will follow the guidelines outlined in the Cochrane Handbook for Systematic Reviews of Interventions. The PRISMA-P checklist is provided in Supplementary File 1.

### Study design

The study is a systematic review which aims to identify the treatment strategies, treatment regimens, treatment durations, treatment success rates, and relapse rates in patients with asymptomatic TB. Both observational studies (cross-sectional studies, case-control studies, and cohort studies) and interventional studies which reported the treatment of patients with asymptomatic or subclinical pulmonary TB will be included.

### Search strategy

We will search Web of Science, MEDLINE, Scopus, Cochrane Library, The Cumulative Index to Nursing and Allied Health Literature (CINHAL), and Embase for original articles, from each database's inception to the date of our final search. No restrictions will be applied regarding publication language. The search strategy combined terms for “asymptomatic”, “tuberculosis”, and “treatment”. Detailed search strategies were provided in Supplementary File 2. Additionally, we will scan the reference lists of included full-text articles and relevant reviews were manually screened for additional eligible studies.

### Eligibility criteria

The eligibility criteria for this review were established based on PICOS typology (population, intervention, comparison, outcomes, and study design). Given the nature of the available evidence, a predefined comparator is not required for inclusion to allow a comprehensive synthesis of treatment outcomes in asymptomatic TB.

### Participants

This review will include individuals diagnosed with asymptomatic or subclinical TB, as defined by the original study authors, regardless of whether they were bacteriologically confirmed or unconfirmed. We will extract and report the operational case definitions used in each study. For the primary analyses, we will focus on studies in which asymptomatic or subclinical TB is supported by at least one objective marker, including microbiological and/or radiographic findings consistent with active TB. Studies defining asymptomatic or subclinical TB solely by the absence of symptoms, without any objective evidence, will still be included but will be analysed and discussed separately. There will be no age restrictions for inclusion.

### Intervention

The intervention of interest is any anti-TB treatment administered to patients with asymptomatic TB.

### Outcome measures

The primary outcome of interest is:

i The proportion of patient with a favourable outcome at the end of follow up, defined as no clear evidence of active TB disease at that time, regardless of treatment strategy. We will adopt the definition of favourable outcome as specified by the original study authors (e.g., treatment success or favourable status).

Additional outcomes include:

i Treatment success rate, defined as the proportion of treated patients who achieved a favourable outcome among all patients receiving TB chemotherapy.
ii Chemotherapy coverage rate, defined as the proportion of patients with asymptomatic TB who received TB chemotherapy.
iii Lost to follow up rate.
iv Mortality rate.
v Adverse events during the treatment.
vi Relapse rate.
vii Anti-TB treatment regimens used.
viii Patient-related outcomes, including tolerance, pill burden, and discontinuation rate by patients themselves.

### Exclusion Criteria

Studies will be excluded if they meet any of the following criteria:

i Review articles, guidelines, case reports, conference abstracts, or in vitro studies
ii Studies lacking sufficient information on the outcomes of interest
iii Studies that do not include patients with asymptomatic TB
iv Studies with overlapping data
v Studies with missing or insufficient data, unless reasonable efforts to obtain additional information from the corresponding authors are successful

This eligibility framework ensures a comprehensive yet targeted selection of studies, facilitating a robust analysis of treatment outcomes in patients with asymptomatic TB.

### Study selection

Two reviewers will independently screen studies based on the inclusion criteria and exclude those meeting the exclusion criteria. Adhering to the PRISMA guidelines, they will minimize selection bias throughout the identification, screening, and inclusion stages. The number of included and excluded studies, along with reasons for exclusion, will be documented from electronic databases and other sources.

Firstly, duplicate records will be removed using EndNote, followed by manual verification to identify and remove any remaining duplicates. Then, the reviewers will screen article titles and abstracts across the databases and other sources to identify potentially eligible studies. Records without sufficient information in the title or abstract will be carried forward to full-text screening to ensure comprehensive assessment.

Full texts of potentially relevant articles will be retrieved and assessed for eligibility based on the PICOS framework to ensure relevance to the target population, intervention types, study design, treatment regimen, and outcomes. Throughout the process, if the full text is unavailable, we will contact the corresponding author for information about the entire article via email. Articles without accessible full texts will be excluded.

Eligible studies meeting all inclusion criteria will be selected for final analysis and synthesis, with the screening process documented in a PRISMA diagram. Any disagreements between the two reviewers will be resolved through discussion and consensus. If needed, a third reviewer will independently verify the title, abstract, and full text to resolve conflicts.

### Data extraction

Two investigators will independently collect relevant data using a predefined form. The following data will be extracted: 1) first author, year of publication, country of study, title, funding source and conflicts of interest; 2) study design, sample size, follow-up period (if applicable), population characteristics, age, gender, HIV status, disease definition, radiographic findings, bacteriological findings, drug resistance pattern; 3) treatment indications, treatment regimen, treatment duration, number of asymptomatic/subclinical TB cases, number of asymptomatic/subclinical TB cases receiving TB chemotherapy; 4) number of patients with a favourable outcome at follow up, treatment success rate or number of cases with treatment success, treatment relapse rate or number of cases with relapse TB, mortality rate or number of deceased cases, number of lost to follow up; 5) safety outcomes (adverse events), any description of tolerance, pill burden, and self-initiated discontinuation rates. If symptomatic cases with TB were included in the study, data for these patients will be extracted in the same manner. When several research used the same database, all relevant articles will be examined to ensure comprehensive data extraction.

### Quality assessment

We will critically appraise included studies using validated tools. Two independent reviewers will assess study quality using the Cochrane Risk of Bias (ROB 2.0) tool for randomized controlled trials [12], or the Newcastle-Ottawa Scale (NOS) for other study designs [13]. In cases of disagreement, a third reviewer will resolve the discrepancy.

### Data synthesis

We will aim to meta-analyze results by outcome, such as success and relapse rates, when possible, using a random-effects DerSimonian-Laird (LD) model to account for between-study heterogeneity [14,15]. We will use an Inverse-variance weighting of studies and visualize pooled results using forest plots created with R software [16]. Statistical heterogeneity will be assessed using the I² statistic. When substantial heterogeneity is observed, studies contributing disproportionately to heterogeneity will be identified and excluded from quantitative synthesis, and meta-analysis will be conducted using the remaining studies where appropriate. Results which we will not be able to meta-analyze will be reported narratively, using the Synthesis Without Meta-Analysis (SWiM) reporting guidelines [17]. Subgroup analyses and meta-regression are not planned. For outcomes with at least 10 included studies, potential publication bias will be assessed using visual inspection of funnel plots.

## Discussion

This systematic review and meta-analysis will explore the treatment regimens and key clinical outcomes reported in existing studies on asymptomatic TB. By synthesizing these data, we aim to gain a clearer understanding of the current treatment landscape for asymptomatic TB and compare its similarities and differences with symptomatic TB within the existing treatment framework.

Efforts to optimize anti-TB treatment have been underway for decades, with significant progress made in recent years. A key advancement has been the growing recognition that a one-size-fits-all approach may be suboptimal, leading to a consensus on the need for stratified assessment based on patients’ baseline characteristics [18,19]. Increasing evidence suggests that some patients with mild TB might be overtreated with standard regimens, while those with more severe TB with high bacterial burden require extended treatment durations. This insight motivates researchers to optimize asymptomatic TB treatment. Although symptom severity does not always correlate directly with disease progression, previous studies indicate that patients with asymptomatic TB typically have a lower bacterial load [9]. As a result, they may be at greater risk of overtreatment with the standard six-month regimen compared to symptomatic patients. This systematic review will summarize the current treatment landscape for asymptomatic TB, providing a critical reference for future efforts to simplify or shorten treatment regimens.

In addition to focusing on treatment success rates, this article will also evaluate the safety of treatment for patients with asymptomatic TB and patient-related outcomes in current studies [20]. For asymptomatic TB, the acceptability of treatment plays a crucial role in influencing patients’ adherence and willingness to complete therapy. This systematic review aims to describe and synthesize existing evidence on patient acceptance and tolerability of treatment, providing insights for future optimization of TB treatment and management.

## Supporting information

S1 File The PRISMA-P checklist. (DOCX)

S2 File Search strategy. (DOCX)
