# Supplementary material for: Treatment of asymptomatic tuberculosis: Protocol for a systematic review of treatment strategies, regimens, and clinical outcomes
Source: PLoS One. 2026 Mar 2;21(3):e0342929. doi: 10.1371/journal.pone.0342929 (PMC12952583; doi:10.1371/journal.pone.0342929)
Supplement: S2 File — (DOCX) [file pone.0342929.s002.docx]

**Review questions**

- What treatment regimen and treatment course have been used for patients with asymptomatic tuberculosis?
- What is the treatment success rate of patients with asymptomatic tuberculosis?
- What is the treatment relapse rate of patients with asymptomatic tuberculosis?

**Search strategy**

- Databases to be searched include: MEDLINE, Embase, Cochrane Library, Scopus, Web of Science, CINAHL databese.
- There will be no language restrictions.
- There are no time limitation.

#1 “subclinic*"[Title/Abstract] OR “asymptomatic*”[Title/Abstract]OR “inapparent” [Title/Abstract] OR “presymptomatic” [Title/Abstract] OR “incipient” [Title/Abstract]

#2 "tuberculosis"[MeSH Terms]

#3 "Tuberculosis"[Title/Abstract] OR "Tuberculoses"[Title/Abstract] OR “Tuberculo*” [Title/Abstract] OR “TB” [Title/Abstract] OR "Koch's Disease"[Title/Abstract] OR "Koch Disease"[Title/Abstract]

#4 #2 OR #3

#5 #1 AND #4

#6 “Therapeutics” [MeSH Terms]

#7 “treatment*” [Title/Abstract] OR “management*” [Title/Abstract] OR “protocol*” [Title/Abstract] OR “regimen*” [Title/Abstract]

#8 #6 OR #7

#9 #5 AND #8

**Study type**

- Observational studies (cross-sectional studies, case-control studies, and cohort studies)
- Interventional studies (trials).

**Population**

This review will include patients with asymptomatic or subclinical pulmonary tuberculosis, regardless of whether they were bacteriologically confirmed or unconfirmed.

**Intervention**

Anti-tuberculosis treatment

**Main outcome**

- Treatment success rate
- Relapse rate

**Secondary outcome**

- Culture conversion rate at 8 weeks after treatment initiation and at the end of treatment
- Median time to sputum culture conversion
- Adverse events during the treatment

**Data extraction**

Data will be extracted using a pre-defined template. The following information will be collected:

- Study Characteristics
- Author(s)
- Year of Publication
- Title
- Journal
- Country/Region
- Funding Source and Conflicts of Interest
- Study Design and Methodology
- Study Design: Specify if it is cross-sectional, cohort, case-control, or interventional study.
- Sample Size: Total number of participants.
- Study Setting: Hospital, community, other.
- Follow-up Period (if applicable)
- Population Characteristics
- Age: Age range and mean age.
- Gender: Male, female, other, not specified.
- HIV Status: Proportion of HIV-positive and HIV-negative participants
- Inclusion Criteria
- Exclusion Criteria
- Diagnostic Methods
- Interventions
- Treatment regimen
- Treatment duration
- Outcomes Data
- Total asymptomatic/subclinical TB Cases
- Treatment success rate or number of cases with treatment success
- Treatment relapse rate or number of cases with relapse TB
- Culture conversion rate or number of cases found to be culture-negative at 8 weeks after treatment initiation and at the end of treatment
- Median time to sputum culture conversion
- Risk of Bias and Quality Assessment
- Additional Notes and Observations
- Limitations Reported by Authors
- Other Relevant Findings
